# Paraneoplastic Diseases of the Central Nervous System

**DOI:** 10.12688/f1000research.21309.1

**Published:** 2020-03-06

**Authors:** Jonathan Galli, John Greenlee

**Affiliations:** 1 Department of Neurology, University of Utah, Salt Lake City, UT, 84108, USA; 2 2. George E. Wahlen Department of Veterans Affairs Medical Center, Salt Lake City, UT, 84148, USA

**Keywords:** neurological, paraneoplastic, antibody-mediated, immunotherapy

## Abstract

Paraneoplastic neurological syndromes are nonmetastatic complications of malignancy secondary to immune-mediated neuronal dysfunction or death. Pathogenesis may occur from cell surface binding of antineuronal antibodies leading to dysfunction of the target protein, or from antibodies binding against intracellular antigens which ultimately leads to cell death. There are several classical neurological paraneoplastic phenotypes including subacute cerebellar degeneration, limbic encephalitis, encephalomyelitis, and dorsal sensory neuropathy. The patient’s clinical presentations may be suggestive to the treating clinician as to the specific underlying paraneoplastic antibody. Specific antibodies often correlate with the specific underlying tumor type, and malignancy screening is essential in all patients with paraneoplastic neurological disease. Prompt initiation of immunotherapy is essential in the treatment of patients with paraneoplastic neurological disease, often more effective in cell surface antibodies in comparison to intracellular antibodies, as is removal of the underlying tumor.

## Introduction

Paraneoplastic neurological syndromes are non-metastatic complications of systemic malignancy, in which clinical syndromes are the result of immune-mediated neuronal dysfunction or death. The syndromes may have their onset as long as five years prior to detection of the associated malignancy, during treatment, or (less often) when the underlying malignancy is in remission. The syndromes occur when the underlying neoplasm expresses proteins that are cross-reactive with neuronal antigens ^1– 4^. Paraneoplastic neurological syndromes are thus among the few autoimmune conditions in which the immune targets that initiate the disease are known. In this review, we concentrate predominantly on paraneoplastic neurological syndromes affecting the central nervous system (CNS) or sensory ganglia; syndromes affecting the myoneuronal junction or peripheral nerves will not be discussed. The article will discuss current concepts of pathogenesis, clinical features of the major paraneoplastic syndromes, and approaches to treatment.

## Pathogenesis

That paraneoplastic neurological injury could have an autoimmune basis was suggested by Wilkinson and Zebrowski in the 1960s ^5^ and Trotter *et al*. in the 1970s ^6^. A definitive association of autoimmunity in paraneoplastic neurological disease came through work by Greenlee and Brashear ^7^ and Jaeckle *et al*. ^8^, who demonstrated antibodies to cerebellar Purkinje cells in patients with paraneoplastic cerebellar degeneration. Over the ensuing years, many other patterns of antibody response have been associated with neurological disease in the presence of other systemic cancers ( Table 1 and Table 2), and it is now recognized that paraneoplastic neurological syndromes may be accompanied by two separate patterns of immune response: syndromes in which the immune response is directed against neuronal receptors or other cell membrane antigens and syndromes in which immunoreactivity is directed against intracellular neuronal proteins. The former group of syndromes are characterized by neuronal dysfunction, may or may not be associated with underlying neoplasia, and are frequently amenable to treatment. Syndromes in the latter group—those in which the immune response targets intracellular antigens—are almost always found in the presence of cancer and are characterized by neuronal death. Neurological deficits in these conditions, once intrinsic neuronal reserve has been exhausted, are irreversible.

**Table 1.** Major paraneoplastic antineuronal antibodies reactive with neuronal membrane antigens.

Antibody	Common neurological phenotypes	Common associated malignancies	Frequency of underlying malignancy	Response to treatment ^a^
Anti-AMPAR	Limbic encephalitis	Breast Lung Thymus	Common	Good in many but not all patients
Anti-LGI1/Anti- CASPR2	Limbic encephalitis Faciobrachial dystonic seizures Morvan’s syndrome	Thymoma (especially in patients positive for both antibodies) Other neoplasms (rare)		Usually poor
Anti-GABAbR	Limbic encephalitis, status epilepticus	Small-cell lung cancer	Common	Poor
Anti-mGluR1	Cerebellar degeneration	Hodgkin’s disease	Common	Good
Anti-mGlur2	Cerebellar degeneration	Small-cell cancer; alveolar rhabdomyosarcoma	Common	Variable
Anti-mGluR5	Limbic encephalitis	Hodgkin’s disease	Common	Good
Anti-VGKC	Cerebellar degeneration (Lambert–Eaton myasthenic syndrome)	Small-cell lung cancer	Common	Good in some patients

Modified from 13. ^a^Treatment involves both immunosuppressive treatment directed against the autoimmune process and treatment of the underlying malignancy.

**Table 2.** Major paraneoplastic antineuronal antibodies reactive with intracellular neuronal antigen.

Antibody	Common neurological phenotypes	Common associated malignancies	Frequency of underlying malignancy	Response to treatment ^a^
Anti-CRMP5	Optic neuritis Cerebellar degeneration Encephalomyelitis	Small-cell lung cancer Breast carcinoma	Common	Poor
Anti-GAD65	Stiff person syndrome Limbic encephalitis Cerebellar ataxia	Thymoma Renal cell carcinoma	Uncommon	Good in stiff person syndrome; otherwise poor
Anti-Hu (ANNA-1)	Limbic encephalitis, encephalomyelitis, dorsal sensory neuropathy	Small-cell lung cancer Neuroendocrine tumors Retinoblastoma (infants)	Almost invariable	Poor
Anti-Ma1	Limbic or brain-stem encephalitis	Non-small-cell lung cancer; other	Almost invariable	Poor
Anti-Ma2	Limbic or brain-stem encephalitis	Testicular or other germ cell tumors Non-small-cell lung cancer	Almost invariable	Good if testicular neoplasm detected and treated
Anti-Ri (ANNA-2)	Cerebellar degeneration, opsoclonus myoclonus, brain-stem encephalitis	Breast Small-cell lung cancer	Almost invariable	Stabilization in some but not all patients
Anti-Tr	Cerebellar degeneration	Hodgkin’s disease	Almost invariable	Poor
Anti-Yo (PCA-1)	Cerebellar degeneration	Ovary, uterus, adnexa Breast	Almost invariable	Poor

Modified from 13. ^a^Treatment involves both immunosuppressive treatment directed against the autoimmune process and treatment of the underlying malignancy.

### The role of tumor antigens in the pathogenesis of paraneoplastic neurological disease

That tumors may harbor antigens cross-reactive with neuronal proteins was demonstrated by early investigators ^1– 3^, and subsequent work demonstrated that these antigens may be detected not only in patients with paraneoplastic neurological disease but also in cancer patients who remain neurologically normal ^9, 10^. More recent studies of ovarian tumors from patients with paraneoplastic cerebellar degeneration and anti-Yo antibodies, the major antibodies associated with the condition, have demonstrated that, although ovarian tumors from anti-Yo–positive and anti-Yo–negative patients were histologically similar, ovarian tumors from anti-Yo–positive patients differ from tumors from patients without anti-Yo antibodies by high rates of genetic alterations in key CDR and, to a greater extent, CDR2L (CDR2-like) Yo antigens and by intense tumor infiltration by plasma cells and cytotoxic CD8 ^+^ lymphocytes ^11, 12^. Recent studies of ovarian teratomas from patients with anti– *N*-methyl-D-aspartate receptor (anti-NMDAR) encephalitis have shown that teratomas from NMDAR encephalitis are much more likely to contain predominantly glial elements suggestive of neuroglial tumors than those without encephalitis and may also have extensive infiltrates of T and B cells as well as IgG and IgA deposits in close contact with the neuroglial components of the teratomas ^14^. All of these more recent data suggest that tumors from patients with paraneoplastic neurological syndromes may have unique properties capable of breaking immune tolerance and triggering a vigorous B- and T-cell response.

### Syndromes associated with antibodies to cell membrane antigens

In this group of conditions, neuronal injury occurs when antibodies bind to and impair the function of neuronal membrane proteins. The best studied of these conditions is anti-NMDAR encephalitis. This condition was initially described in women with ovarian teratomas but may also occur in the absence of underlying neoplasia and can also affect males. In anti-NMDAR encephalitis, antibody binding to the NMDAR results in receptor cross-linking internalization and in depletion of receptors on the cell surface ^15– 17^. Similar immunological mechanisms are thought to be shared by conditions associated with antibodies to the α-amino-3-hydroxy-5-methyl-4-isoxazolepropionic acid (AMPA) receptor and many but not all other antibodies to cell surface antigens ^18– 20^. An important point in these conditions is that reconstitution of neuronal surface receptors may occur slowly and may require protracted therapy over weeks to months ^21^.

### Syndromes associated with an immune response to intracellular neuronal proteins

The mechanisms of neuronal injury in this group of conditions have remained controversial and understanding of the role of these antibodies in disease pathogenesis has been greatly hindered by the lack of an animal model. Early attempts to produce paraneoplastic disease in animals by passive transfer of antibodies or immunization with antigen or nucleic acids were uniformly unsuccessful ^22– 25^, leading many investigators to conclude that the associated antibodies were simply markers for underlying neoplasia. T lymphocyte response in patients with antibodies to internal cellular proteins (for example, Yo and Hu) has been repeatedly demonstrated: infiltrates of cytotoxic (CD8 ^+^) lymphocytes have been detected in brains of patients with encephalomyelitis and anti-Hu antibody ^26^ and in cerebrospinal fluid (CSF) of a patient with anti-Yo–associated paraneoplastic cerebellar degeneration ^27^. Cross-reactive T-cell receptors have been reported in brain and tumor tissue from a patient with paraneoplastic encephalomyelitis ^28^. Interestingly, however, Sillevis Smitt *et al*. failed to detect cytotoxic T lymphocytes in serum or CSF of patients with anti-Hu antibodies and paraneoplastic neurological disease ^29– 31^. We are aware of only one attempt to cause neuronal death using T cells sensitized to paraneoplastic antigens in an animal model. That study, by Pellkofer *et al*., though producing a predominantly meningitis response, did not duplicate paraneoplastic neurological disease ^32^.

The role of antibodies in causing neuronal injury is controversial. Neurons have traditionally been considered to exclude immunoglobulins (IgG); for this reason, it has been thought unlikely that antibodies could reach their intracellular antigenic targets. However, the ability of neurons in living animals to incorporate immunoglobulins was demonstrated many years ago by Fabian and Petroff ^33^ and was suggested by Griffin *et al*. ^34^ in experimental Sindbis virus infection. Neuronal uptake of paraneoplastic and other IgGs was documented by Graus *et al*. ^23^ in studies using intraventricular infusion of IgG in guinea pigs and by Greenlee *et al*. ^35^ following blood–brain barrier disruption after intraperitoneal injection of anti-Yo IgG. Both our laboratory and that of Vedeler *et al*. have demonstrated that anti-Yo IgG can be taken up by Purkinje cells in rat cerebellar slice cultures and can cause cell death ^36– 39^; and anti-Hu IgG, associated with encephalomyelitis in the setting of small-cell lung cancer, has been shown to produce neuronal death in dispersed cultures of cerebellar neurons and of multiple neuronal populations in slice cultures of rat brains ^40, 41^. These studies have not been extended to living animals, however, and a major challenge in developing such a model has been the difficulty of achieving antibody penetration across the blood–brain barrier into brain over prolonged periods of time. Study of a third possible mechanism of injury—that paraneoplastic neuronal injury involves both T and B cells—must also await the development of an animal model.

## Clinical presentations of major paraneoplastic neurological syndromes

### Anti-NMDAR encephalitis

Anti-NMDAR encephalitis was initially described in women with ovarian teratomas but can occur in patients with other (predominantly germ cell) neoplasms and also in both females and males without neoplasia ^4, 21^. It can also occur as apparent clinical relapse in patients recovering from herpes simplex virus encephalitis ^42^. Anti-NMDAR encephalitis remains by far the most commonly diagnosed disorder associated with antineuronal autoantibodies and its frequency surpasses those of individual viral encephalitides ^43^. The disorder is complex, and a range of symptoms suggests the involvement of multiple brain regions and can include cognitive and memory deficits, refractory seizures (including status epilepticus), significant dysautonomia, limbic symptoms, psychiatric disturbances (including agitation paranoia), catatonia, movement disorders, seizures, autonomic dysfunction, hypoventilation which may require artificial ventilation, and coma ^21, 44– 46^. Patients often present initially with behavioral changes, including insomnia, agitation, paranoia, and hallucinations, developing over several weeks to months ^47^. Magnetic resonance imaging (MRI) often demonstrates T2 hyperintensity within the mesial temporal lobes ^48^. An electroencephalogram (EEG) may demonstrate temporal slowing or epileptogenic activity ^49^. Very few cases of NMDAR encephalitis have come to autopsy but these suggest some degree of neuronal loss and extensive brain infiltration by lymphoid cells, in particular by B cells and plasma cells. The disorder is often responsive to immunomodulatory therapy, including steroids, plasma exchange, intravenous immunoglobulin (IVIG), and rituximab. Recovery, however, may require prolonged treatment over weeks to months, and some patients can relapse.

### Autoimmune encephalitis with antibodies to the voltage-gated potassium channel complex: LGI1 and CASPR2

Autoimmune encephalitis associated with antibodies to LGI1 and CASPR2 is only infrequently associated with underlying malignancy; however, in patients positive for both antibodies, the likelihood of cancer approaches 44% and thymomas constitute the majority of these cases ^50^. Anti-LGI1 encephalitis is typically characterized by faciobrachial dystonic episodes that may or may not have an ictal EEG accompaniment. Anti-CASPR2 encephalitis was initially associated with Morvan’s syndrome. Both entities, however, may have much more complex central and peripheral semiology, including seizures, psychiatric or personality changes, autonomic instability, and peripheral neuropathic symptoms. Thirty-nine percent of patients with anti-LGI1 antibodies will exhibit hyponatremia. As with anti-NMDAR encephalitis, response to immunomodulatory treatment is often excellent, but treatment may need to be prolonged.

### Subacute (paraneoplastic) cerebellar degeneration

Paraneoplastic cerebellar degeneration is characterized by remorselessly progressive signs of cerebellar injury, including central and appendicular ataxia, vertigo, and nystagmus. As the disease progresses, the severity of cerebellar deficits is often well beyond that of most other cerebellar syndromes, leaving the individual unable to sit, stand, or even speak ^51^. MRI brain in these patients may be initially normal but later may demonstrate cerebellar atrophy ^52^. Major associated tumors include gynecological and breast malignancies, small-cell lung cancer, and Hodgkin’s disease. Paraneoplastic cerebellar degeneration should be considered as a diagnosis in any adult patient with subacute progressive cerebellar ataxia in the absence of a family history.

Paraneoplastic cerebellar degeneration may be accompanied by any of several well-described antibodies, and the most common include anti-Yo (PCA-1), PCA-2, anti-Hu (ANNA-1) anti-Ri (ANNA-2), and anti-Tr ^52^. Rare cases may be associated with antibodies to the metabotropic glutamate receptors, mGluR1 and mGluR2 ^53, 54^. The antibody is often suggestive of the underlying tumor, with anti-Yo most often associated with breast or gynecological malignancies, anti-Ri associated with breast or lung malignancies, anti-PCA-2 with lung malignancies ^55– 57^, and anti-Tr and anti-mGluR1 with Hodgkin’s disease ^53, 58, 59^. Cerebellar symptoms in patients with anti-Ri and anti-Hu antibodies may be accompanied by opsoclonus. With the exception of cases associated with anti-mGluR1 and possibly those associated with anti-Ri, the course of disease is characterized by progressive, irreversible neuronal loss, making early treatment of great importance and limiting response to immunotherapy as the disease progresses ^60^.

### Paraneoplastic encephalomyelitis

Encephalomyelitis refers to inflammation of the brain or spinal cord (or both) and may present with cortical, brain stem, or spinal involvement either in isolation or concomitantly. Discussed below are specific presentations that clinicians should be aware of while remaining cognizant that one or more may be present at the same time. Anti-NMDAR encephalitis is discussed above.

***Limbic encephalitis.*** Encephalitis involving primarily the limbic system is characterized by a constellation of symptoms and signs that may include personality changes, irritability, depression, seizures, memory loss, and sometimes dementia ^47, 61^. Limbic encephalitis has been associated with many different antineuronal antibodies. Anti-Hu and anti-Ma2 antibodies have well-described associations with small-cell lung cancer and testicular germ cell tumors, respectively ^62, 63^. Anti-mGluR5 antibodies may be associated with limbic encephalitis in Hodgkin’s disease ^58^. Anti-GABAbR and anti-AMPAR antibodies are most commonly associated with small-cell lung cancer and breast cancer or thymoma, respectively ^64, 65^. Other antibodies that may be associated with limbic encephalitis include those to amphiphysin, Caspr2, LGI1, and GAD65: these may have associated malignancies but, except in cases where antibodies to Caspr2 and LGI1 are present together, more frequently occur in the absence of malignant disease ^66– 71^.

***Brain-stem encephalitis.*** Brain-stem involvement in paraneoplastic disease may present with varying phenotypes, depending on the region of the brain stem that is involved. Gaze palsies present in midbrain involvement, facial palsy or vertical gaze palsies present in pontine involvement, dysarthria or dysphagia or central hypoventilation present in medullary involvement have been documented; patients may also have pyramidal symptoms and gait impairment ^62, 72^. Anti-Hu and Ma2, associated most commonly with small-cell lung cancer and testicular tumors, respectively, should be strongly considered. Immunotherapy may stabilize symptoms to some degree; however, improvement is often limited, emphasizing the importance of early diagnosis and treatment ^62, 72, 73^. One peculiar syndrome is opsoclonus myoclonus syndrome, which is characterized by rapid and jerking eye movements that are accompanied by myoclonic movements of the extremities and ataxia ^74^. Serologically, anti-Hu and anti-Ri antibodies are most commonly associated with this syndrome in adults ^75, 76^. In children, neuroblastoma is the most frequently associated malignancy ^77^. Recently, Kelch-like protein 11 antibodies were identified in patients with brain-stem and cerebellar symptoms and associated testicular seminomas ^78^.

***Myelitis.*** Paraneoplastic myelitis should be considered in patients presenting with subacute myelopathy. Presenting symptoms may be non-specific and consistent with myelitis, including flaccid or spastic paraparesis or tetraparesis, urinary or bowel dysfunction, or sensory loss. Rarely, cancer may be accompanied by involvement of spinal cord motor neurons to produce a syndrome suggestive of amyotrophic lateral sclerosis ^79^. Paraneoplastic myelitis most commonly occurs in patients with underlying small-cell lung or breast neoplasms. CSF analysis may demonstrate pleocytosis, elevated protein, or the presence of oligoclonal bands with imaging classically demonstrating longitudinally tract-specific enhancement but can be normal in some patients ^80^. Paraneoplastic myelitis is most frequently associated with anti-Hu antibodies, and fewer cases are associated with anti-Ri or anti-CRMP5 ^79, 81– 83^. Necrotizing myelitis has also been described; it has a more fulminant presentation, which can occur without associated paraneoplastic antibodies ^84^.

### Dorsal sensory neuropathy

Dorsal root ganglionopathies typically present with subacute, asymmetrical, often painful progressive sensory loss usually affecting proprioception and vibratory sensation in comparison with temperature and discriminatory touch ^85^. There may also be an association with additional autonomic, cerebral, or cerebellar symptoms ^86^. CSF analysis often demonstrates pleocytosis or the presence of oligoclonal bands, and nerve conduction studies are particularly helpful in localizing involvement in the dorsal root ganglia rather than peripheral nerves ^87, 88^. Dorsal sensory neuronopathy may occur in the setting of small-cell lung cancer, and patients will often have anti-Hu antibodies ^89, 90^. Dorsal sensory neuronopathy may also occur in non-cancerous disorders such as Sjögren syndrome ^89^.

## Evaluation and diagnosis

The clinical presentation of paraneoplastic neurological disease varies widely and often requires a high degree of clinical suspicion in order not to miss the diagnosis. Although the onset of symptoms can be very rapid, most patients develop symptoms in a subacute manner over weeks or sometimes months. Patients may have coexisting autoimmune disease, a family history of autoimmunity or cancer, or risk factors for malignancy. CSF evaluation is essential in these patients. In many patients, CSF analysis may demonstrate pleocytosis, elevated protein, or the presence of oligoclonal bands or a combination of these. In some patients, however, normal CSF does not exclude the possibility of paraneoplastic neurological injury ^91^. As the clinical presentation of a specific antibody can vary widely, our recommendation is to test with antibody panels, which are commercially available, rather than single-antibody testing ^92^. There is frequently a time lag before results of antibody testing become available. For this reason, prompt empiric therapy should be considered, especially in patients with rapidly worsening syndromes.

In patients with suspected paraneoplastic neurological disease, screening for underlying malignancy is mandatory. Positron emission tomography (PET) is more sensitive to smaller tumors than computed tomography. Antineuronal antibodies may act as markers for specific tumors such as breast or reproductive organ tumors for anti-Yo, small-cell lung cancer for anti-Hu, breast or lung for anti-Ri, or testicle for anti-Ma2. Additional testing, including mammography and pelvic or testicular ultrasound, may be necessary. If malignancy is identified with imaging, treatment should be guided with the aid on an oncologist. Guidelines for diagnosis and tumor search are available ^93^.

## Treatment

Prompt initiation of treatment in paraneoplastic disease—especially where the condition is accompanied by antibodies to intracellular antigens—is essential to prevent substantial permanent neurological impairment or death. As testing may take several days to return, we recommend empiric treatment while awaiting results in patients who are highly suspicious for paraneoplastic disease. Subacute onset, focal neurological deficits, abnormal T2 hyperintensity in the temporal lobes on MRI, and inflammatory CSF are suggestive of an underlying antibody-mediated etiology and should prompt the provider to consider immunotherapy ^94^. Although there are no controlled trials to date to guide therapy decisions in paraneoplastic disease, many therapeutic approaches have been employed. Treatment is thus empiric and should be based on the severity of the underlying pathological antibody, the patient’s condition, co-morbid medical conditions, and response to immunotherapy.

Treatment of paraneoplastic neurological syndromes begins with therapies addressing the underlying malignancy if one is known or attempts to identify and treat a malignancy not yet detected. Response to treatment of the neurological disorder differs between syndromes associated with antibodies to cell membrane antigens, such as anti-NMDAR encephalitis, and syndromes associated with antibodies reactive with intracellular antigens, such as anti-Yo or anti-Hu. Paraneoplastic neurological syndromes associated with antibodies to cell membrane antigens, such as anti-NMDAR, frequently exhibit a good response to treatment. Intravenous methylprednisolone, usually given as 1000 mg daily for 3 to 5 days, is often used as first-line therapy ^95^. Methylprednisolone is often combined or with or followed by treatment with plasma exchange or IVIG or both. Plasma exchange may be carried out using exchanges on an every-other-day basis for a total of five to seven sessions ^96^. IVIG is typically dosed at 2 g/kg over three to five days ^97^. Clinicians should be aware that performing plasmapheresis following IVIG administration will remove the therapeutic IgG just administered. For this reason, plasma exchange should always be performed prior to IVIG. Patients failing these modalities should be considered for treatment with rituximab, typically administered as 1000 mg intravenously for two doses 14 days apart followed by every-six-months dosing, or cyclophosphamide, typically dosed 500 to 1000 mg/m ^2^ every month ^98, 99^. Also crucial to the long-term treatment in these patients are the identification and treatment of their underlying malignancy ^100^.

Treatment of paraneoplastic neurological disease involving antibodies against intracellular antigens such as anti-Hu, anti-Yo, and anti-Ri tends to be more difficult. Corticosteroids, plasma exchange, IVIG, rituximab, and cyclophosphamide have all been used with varying—and usually poor—degrees of success ^13^. In these conditions, irreversible neuronal loss occurs over time, making prompt or even urgent treatment extremely important. Evidence suggests that patients may have a better prognosis with earlier initiation of treatment, and pathological studies clearly demonstrate that neuronal loss occurs over time ^101, 102^. Although treatment may induce only partial remission of neurological symptoms, prompt initiation of therapy may still allow significant functional improvement ^13, 103, 104^. Similar to syndromes associated with antibodies against neuronal membranes, intravenous methylprednisolone is often used as initial treatment, with consideration of concomitant treatment with IVIG in more severe cases Plasmapheresis is a reasonable strategy to consider, although the paraneoplastic antibody may not be removed from the CNS and patients often do not respond to treatment ^13^. Both cyclophosphamide and rituximab have been used with some benefit in paraneoplastic disorders, although neither treatment has demonstrated consistent response or decreasing titers of antibody ^13, 60, 105, 106^. One difficulty in determining the effect of different treatment modalities in these conditions is that, in most reports, therapy has been instituted late in the disease course, at a time when neuronal injury and death are already extensive. Importantly, treatment of the underlying tumor is imperative to preventing additional progression of the neurological syndrome associated with the paraneoplastic antibody ^13, 106^.

Treatment of any paraneoplastic neurological disorder should be closely coordinated with an experienced care team, including an oncologist, neurologist, and surgeon if necessary. Treatment decisions regarding immunotherapy should be agreed upon within the care team, taking into account the timing of necessary radiation or chemotherapy and its effect on timing of immunotherapy. Prompt treatment of the underlying malignancy is important in the overall patient outcome.
